# Highly Selective H_2_S Gas Sensor Based on Ti_3_C_2_T_*x*_ MXene–Organic Composites

**DOI:** 10.1021/acsami.2c19883

**Published:** 2023-01-25

**Authors:** Seyed Hossein Hosseini-Shokouh, Jin Zhou, Ethan Berger, Zhong-Peng Lv, Xiaodan Hong, Vesa Virtanen, Krisztian Kordas, Hannu-Pekka Komsa

**Affiliations:** † Microelectronics Research Unit, Faculty of Information Technology and Electrical Engineering, University of Oulu, P.O. Box 4500, FIN-90014Oulu, Finland; ‡ Department of Applied Physics, Aalto University, FIN-00076Aalto, Finland; § Research Unit of Medical Imaging, Physics and Technology, Faculty of Medicine, University of Oulu, Aapistie 5A, 90220Oulu, Finland

**Keywords:** MXene, Ti_3_C_2_T_x_, H_2_S sensing, surface functional groups, density functional theory

## Abstract

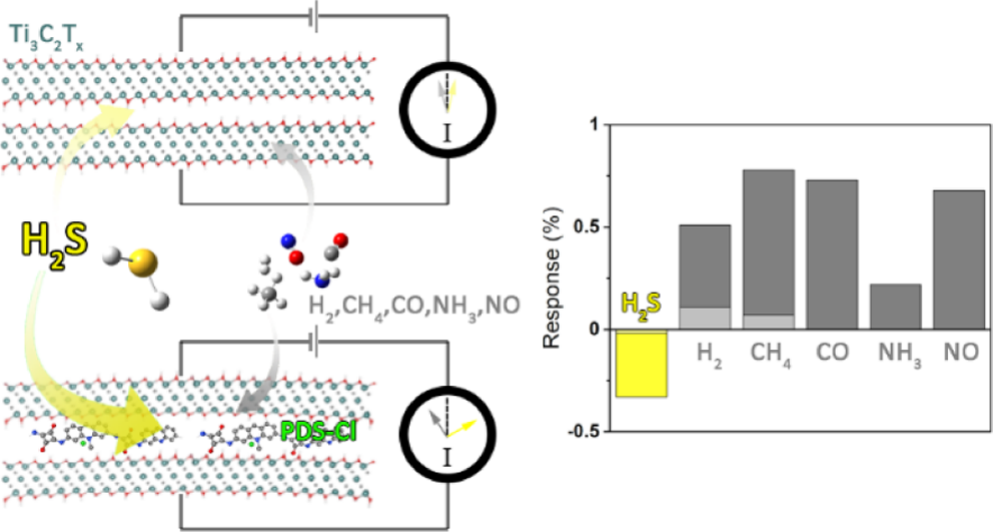

Cost-effective and high-performance H_2_S sensors are required for human health and environmental monitoring. 2D transition-metal carbides and nitrides (MXenes) are appealing candidates for gas sensing due to good conductivity and abundant surface functional groups but have been studied primarily for detecting NH_3_ and VOCs, with generally positive responses that are not highly selective to the target gases. Here, we report on a negative response of pristine Ti_3_C_2_T_*x*_ thin films for H_2_S gas sensing (in contrast to the other tested gases) and further optimization of the sensor performance using a composite of Ti_3_C_2_T_*x*_ flakes and conjugated polymers (poly[3,6-diamino-10-methylacridinium chloride-*co*-3,6-diaminoacridine-squaraine], PDS-Cl) with polar charged nitrogen. The composite, preserving the high selectivity of pristine Ti_3_C_2_T_*x*_, exhibits an H_2_S sensing response of 2% at 5 ppm (a thirtyfold sensing enhancement) and a low limit of detection of 500 ppb. In addition, our density functional theory calculations indicate that the mixture of MXene surface functional groups needs to be taken into account to describe the sensing mechanism and the selectivity of the sensor in agreement with the experimental results. Thus, this report extends the application range of MXene-based composites to H_2_S sensors and deepens the understanding of their gas sensing mechanisms.

## 1 Introduction

Hydrogen sulfide (H_2_S) is a toxic and flammable gas, largely found in petroleum and mining industries as well as in our daily life, e.g., putridity of foods and bacterial breakdown of human and animal wastes. Exposure to H_2_S for humans has the risk of causing severe health problems such as eye and throat injury, dizziness, and loss of sense of reasoning at low concentrations, and it can even lead to death at a very high concentration (above 1000 ppm). According to the UK Health and Safety Executive standard, the short-term (about 8 h) exposure limit is 5 ppm.^1^ Hence, the quality, performance, and accuracy of the detection sensors are very crucial. Most of the available H_2_S gas sensors, however, are expensive and suffer from various problems such as high cost and power consumption, the high limit of detection (LOD), low selectivity, and inflexibility. Therefore, developing a readily available and low-cost gas sensor with better selectivity and LOD toward H_2_S is necessary for human health and environmental monitoring.

Recently, two-dimensional (2D) materials (e.g., graphene,^2^ MoS_2_,^3^ black phosphorus,^4^ and MXene^5^) have attracted intensive research interest due to their unique physical and chemical properties, such as the large surface area, versatile surface chemistry, and room-temperature gas sensing capability.^6^ Especially MXenes, consisting of 2D transition-metal carbides and nitrides, have shown promise for gas sensors due to outstanding metallic conductivity (10^3^–10^4^ S cm^–1^), high mechanical stability, high hydrophilicity, and abundant surface chemistry for gas adsorption.^6^ These layered materials have a universal formula of M_*n*+1_X_*n*_T_*x*_, where M stands for early transition metals (Ti, V, Nb, Ta, Cr, Mo, etc.), X represents carbon and/or nitrogen, T_*x*_ denotes the hydrophilic surface functional groups, such as =O, −OH, or −F, and *n* = 1–3.^7,8^ Lee et al. and Kim et al. were among the first to investigate gas sensing performance of pristine MXenes,^9,10^ followed by a rapidly increasing number of studies.^11−15^ In particular, the surface functional groups of MXenes provide a hydrophilic surface with highly negative zeta potentials, in the range of −30 to −80 mV, which facilitates efficient processing of hybrid MXene structures with organic polymers (with charged end-groups) in aqueous environment in contrast to other 2D materials. Forming composites of MXenes with organic materials has been already shown to be a feasible strategy to enhance both sensitivity and selectivity of gas sensors.^16−18^ For instance, PEDOT:PSS/MXenes^6^ and cationic polyacrylamide/MXene composites^19^ showed a high response to NH_3_, 36% at 100 ppm and 40% at 2000 ppm, respectively. However, despite the promising results, MXene-based gas sensors are still in their infancy and limited to sensors with a small response, constrained detection diversity with usually poor selectivity to the target gas. For example, to the best of our knowledge, only one H_2_S gas sensor and one electrochemical H_2_S sensor based on the Ti_3_C_2_T_*x*_-related materials have been reported thus far^20,21^ in which the sensing response for the gas sensor was mainly attributed to the Ag nanoparticles. Moreover, the intriguing role of intrinsic surface functional groups in the gas sensing performance has not been evaluated extensively even with theoretical calculations, which impairs the understanding of the sensing mechanism.

In this work, we investigate the gas-sensing performance of pristine Ti_3_C_2_T_*x*_ and its nanocomposites with poly[3,6-diamino-10-methylacridinium chloride-*co*-3,6-diaminoacridine-squaraine] (PDS-Cl). While we observe clear H_2_S selectivity (negative response) already on the pristine thin film of Ti_3_C_2_T_*x*_ sensors, the composites of PDS-Cl polymer and Ti_3_C_2_T_*x*_ (Ti_3_C_2_T_*x*_/PDS-Cl) retain excellent selectivity toward H_2_S and provide a higher surface to volume ratio for MXene flakes that consequently enhances the sensing response (∼30 times higher compared to pristine MXene at 1 ppm H_2_S) with low detection limit (0.5 ppm) and good repeatability. To gain detailed insights into the interaction between gas molecules and Ti_3_C_2_T_*x*_, we carried out density-functional theory (DFT) calculations, where we accounted for the fact that MXene surfaces contain a mixture of =O, −OH, and −F functional groups. We show that this has a dramatic effect on gas adsorption (charge transfer and adsorption energy) and is necessary for reproducing the experimental observations. Based on these, we finally propose a sensing mechanism.

## 2 Experimental Section/Methods

### 2.1 Synthesis of Ti_3_C_2_T_*x*_

Aqueous dispersion of Ti_3_C_2_T_*x*_ was synthesized using the MILD method with minor modification.^22^ In a typical synthesis, Ti_3_AlC_2_ (2 g, 325 mesh, Carbon-Ukraine) was added gradually to a stirring mixture of 40 mL 9 M HCl and 2 g LiF (2 g, 325 mesh, Sigma-Aldrich) at 35 °C. After 24 h, the product was separated by a centrifuge and washed with deionized water (DI) until pH > 5. 40 mL water was then added to the sediment and vortexed for 30 min. The supernatant containing few and multiple layered Ti_3_C_2_T_*x*_ was obtained by centrifuging the mixture at 3500 rpm for 15 min and then storing at 4 °C before use. The concentration was measured by weighing a vacuum dried self-standing film of certain volume of the Ti_3_C_2_T_*x*_ dispersion.

### 2.2 Synthesis of PDS-Cl

Acriflavine (230 mg, Sigma-Aldrich) and squaric acid (114 mg, Sigma-Aldrich) were dissolved in 15 mL pyridine and 35 mL *n*-butanol, respectively. The two solutions were mixed and then refluxed and stirred at 120 °C for 16 h under N_2_ protection. After being cooled to room temperature, the mixture was filtered and washed using CH_2_Cl_2_, CH_3_OH, and saturated NaCl aqueous solution. The obtained PDS-Cl was dried in an oven at 80 °C for 24 h. The product was collected as a dark brown powder.

### 2.3 Ti_3_C_2_T_*x*_/PDS-Cl Composite Preparation

**Figure 1:**
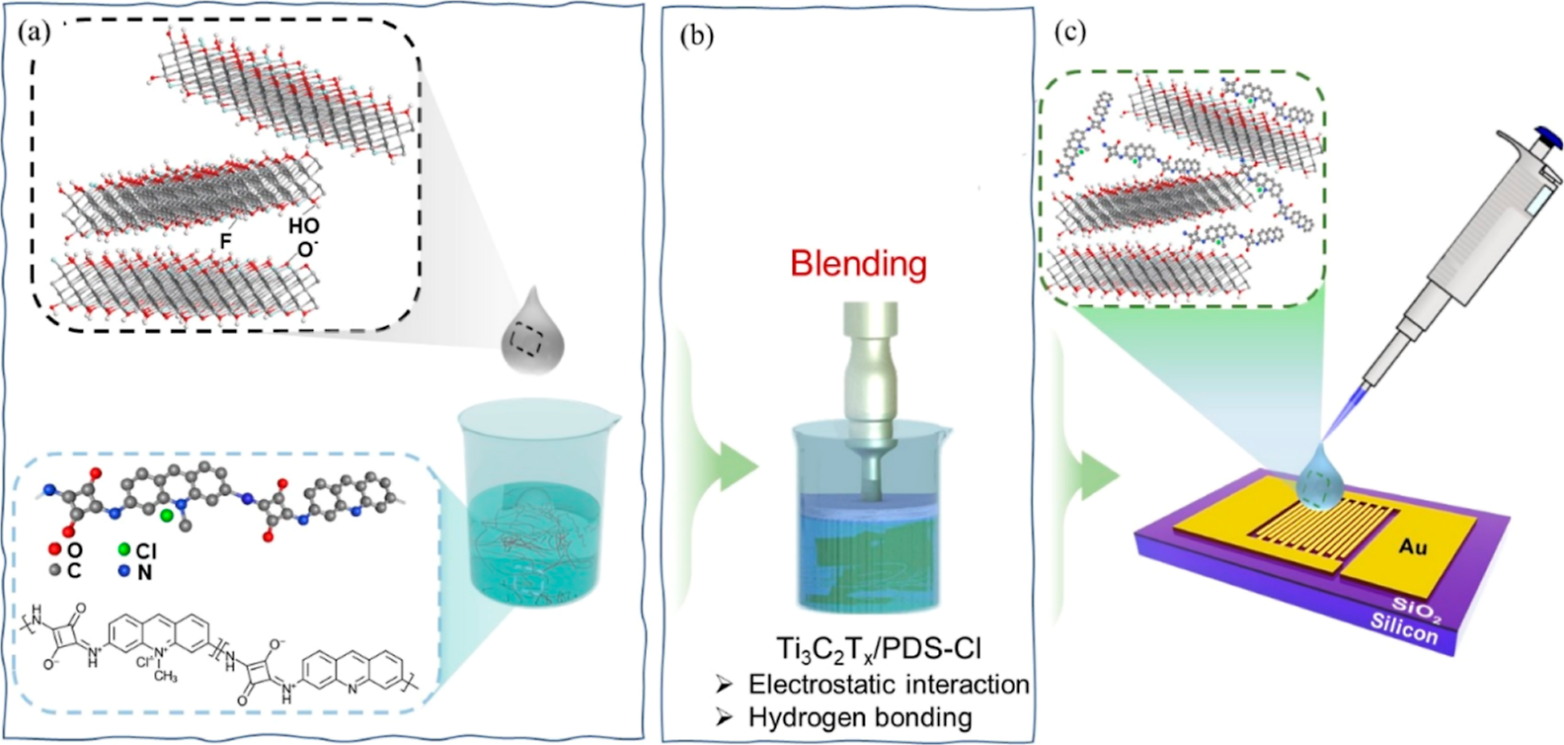
Schematic illustration of Ti_3_C_2_T_*x*_/PDS-Cl composite synthesis and sensor fabrication: (a) after dissolving PDS-Cl polymer in water, the MXene ink was added to the polymer solution. (b) Tip sonication (for 2 min at 20 W) was utilized to facilitate the physical blending. (c) 1 μL of composite solution was drop-casted on prepatterned Au electrodes to fabricate the gas sensors.

We synthesized poly[3,6-diamino-10-methylacridinium chloride-*co*-3,6-diaminoacridine-squaraine] (PDS-Cl), composited with Ti_3_C_2_T_*x*_ through a facile in situ physical blending, as shown in Figure 1a,b. The weight percentage is calculated using eq 1
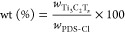
1where  and *w*_PDS-Cl_ are the weights of the Ti_3_C_2_T_*x*_ and PDS-Cl, respectively. Table S1 shows the required mass ratio of Ti_3_C_2_T_*x*_ and PDS-Cl for samples with different wt % of Ti_3_C_2_T_*x*_.

For instance, to prepare the Ti_3_C_2_T_*x*_/PDS-Cl with a mass ratio of 4 wt %, 1 mg PDS-Cl was dispersed in 1 mL DI water by bath sonication for 30 min. After that, 3.3 μL of Ti_3_C_2_T_*x*_ aqueous ink (C = 12 mg•mL^–1^) was added to the solution followed by 2 min of tip sonication at 20 W to achieve uniform distribution of Ti_3_C_2_T_*x*_. The same procedure was repeated for other mass ratios by changing the volume of Ti_3_C_2_T_*x*_ aqueous ink.

### 2.4 Characterization

X-ray photoelectron spectroscopy (XPS) measurements were performed with a Thermo Fisher Scientific Escalab 250 XI system with an Al Kα source. Raman spectra were performed by a Thermo Scientific DXR2xi Raman imaging microscope (excitation wavelength, λ = 785 nm). The microstructure of synthesized material was studied by field-emission scanning electron microscopy (FESEM, Zeiss ULTRA plus and equipped with EDX), transmission electron microscopy (TEM, JEOL JEM-2200FS EFTEM/STEM 200 kV), and energy-dispersive X-ray elemental mapping. Fourier transform infrared spectroscopy (FTIR) of MXene–polymer hybrid was performed on a Spectrum Two FT-IR spectrometer with an ATR model (PerkinElmer, UK). The X-ray diffraction (XRD) was carried out by Rigaku Smart Lab 9 kW, Cu Kα-radiation with a 0.02° of step width. Dynamic light scattering (DLS) data were obtained using a Zeta sizer Nano ZS 90.

### 2.5 Sensor Fabrication and Gas Sensing Setup

To fabricate sensors, 1 μL DI dispersion (1 mg•mL^–1^) of the Ti_3_C_2_T_*x*_/PDS-Cl composite was drop-casted on the Si/SiO_2_ substrate (4 × 6 × 0.5 mm), with 25 pairs of Au–Ti interdigitated electrodes (electrode distance and width were both 20 μm), to form a sensitive film and dried at room temperature, as shown in Figure 1c.

The sensing performance of materials was studied in a Linkam THMS600 heating and freezing stage connected to an Agilent 3458A multimeter at 1 V of constant bias, which are shown in Figure S1. Different concentrations of NH_3_, NO, H_2_S, CH_4_, CO, and H_2_ were obtained by LabView driven mass flow controllers. Nitrogen gas (N_2_) and dry air were used as the carrier gas to dilute these gases to the desired concentrations, while the operating temperature was maintained at room temperature (30 °C). The total gas flow rate was kept constant at 100 mL min^–1^ in all experiments except for selectivity that it was 500 mL min^–1^. To adjust the relative humidity level, an airflow was bubbled through a water-containing flask and then diluted with N_2_ gas before introducing it into the test chamber. The ultimate humidity of the test gas was calibrated via a commercial humidity sensor.

### 2.6 Computational Methods

All DFT calculations were performed using cp2k software.^23−25^ The PBEsol functional^25^ was used with a Goedecker–Teter–Hutter (GTH)^26,27^ pseudopotential and the Gaussian-type basis set MOLOPT.^28^ Van der Waals interactions were accounted by adding the D3 dispersion correction.^29^ A cutoff energy of 800 Ry was set for the expansion of the electron density in plane waves. Geometry optimizations were performed on 4 × 4 × 1 supercell using a 3 × 3 × 1 Monkhorst–Pack *k*-point grid. An out-of-plane lattice constant of 30 Å was used to provide a sufficiently large vacuum region (MXene thickness as calculated from the outermost H atoms is about 9.2 Å). The other lattice parameters were optimized for both mixed and fully O-terminated surfaces and the optimal values were then used and kept constant when adding the gas molecules. The charge transfer was computed using the Mulliken charges as directly implemented in cp2k. We defined it as the difference between the total MXene charge in the presence of and without the gas molecule. This means that a positive charge transfer represents electrons going from the molecule to the MXene layer. The adsorption energy *E*_a_ was defined as , where *E*_ads_, *E*_MXene_, and *E*_mol_ are the energies of the MXene layer with the adsorbed molecule, the pristine MXene layer, and the isolated gas molecule, respectively. The adsorption energy and charge transfer calculated without the D3 dispersion correction are listed in Table S2.

## 3 Results

### 3.1 Material Characterization

We carried out material characterization for both pristine Ti_3_C_2_T_*x*_ and its polymer nanocomposites. The polar charged structure of squaraine in PDS-Cl provides stronger electrostatic interactions between polymers and the Ti_3_C_2_T_*x*_ flakes,^30^ and it can offer ion–dipole interactions or hydrogen bonding with MXenes, forming numerous heterojunction interfaces that may benefit the gas-sensing properties of the composite. For instance, DLS data in Figure S2 show that increasing the MXene content gradually increases the average particle size from 371 nm in the pristine MXene to 4317 nm in PM-20. Such soft aggregation is caused by the electrostatic interaction between negatively charged MXene flakes and the positively charged quaternary ammonium cations in the PDS-Cl.^31^

**Figure 2:**
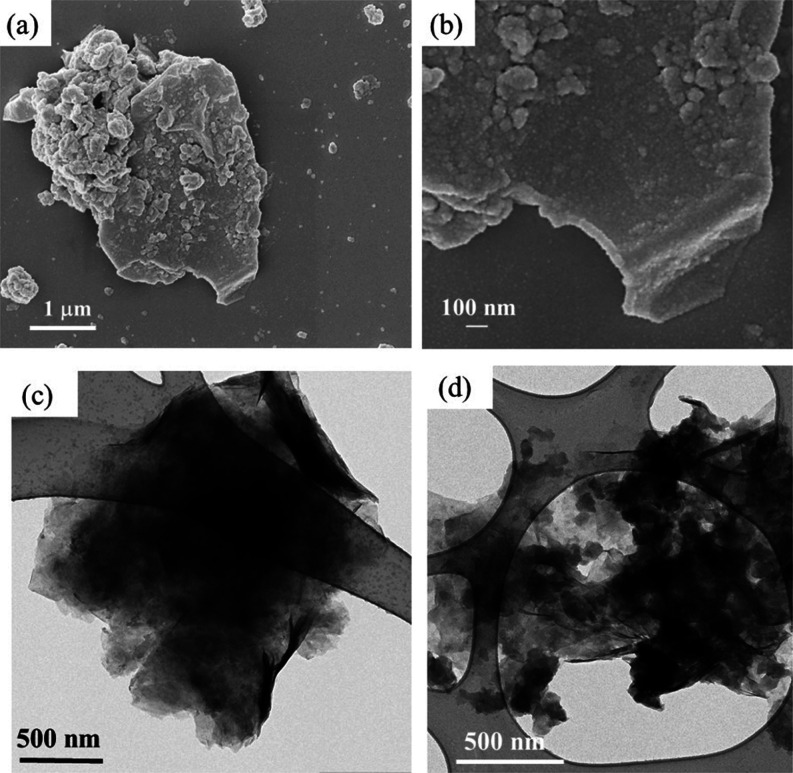
Microstructure analysis: (a) SEM and (b) high-resolution SEM images of samples with 10 wt % of MXene. TEM image of (c) pristine Ti_3_C_2_T_*x*_ flakes and (d) composite sample with 10 wt % of the MXene.

Scanning electron microscopy (SEM) images of the composite, as shown in Figure 2a,b, show the heterojunction interfaces and the layered structures of Ti_3_C_2_T_*x*_ flakes. This layered structure is also evident from the TEM analysis of pristine MXene, as shown in Figure 2c. TEM images of pristine Ti_3_C_2_T_*x*_ and the composites (Figure 2c,d, respectively) indicate a successful blending of the MXene and polymer even for very thin samples, which is further supported by energy-dispersive X-ray (EDX) imaging that provides the elemental distribution of the composite structure, as shown in Figure S3. Pristine MXene sheets should be free of N and Cl but present in the polymer, whereas the opposite is true for Ti. Moreover, EDX indicates the presence of fluorine atoms that probably originate from the functional groups at the surface of the Ti_3_C_2_T_*x*_ flakes.

**Figure 3:**
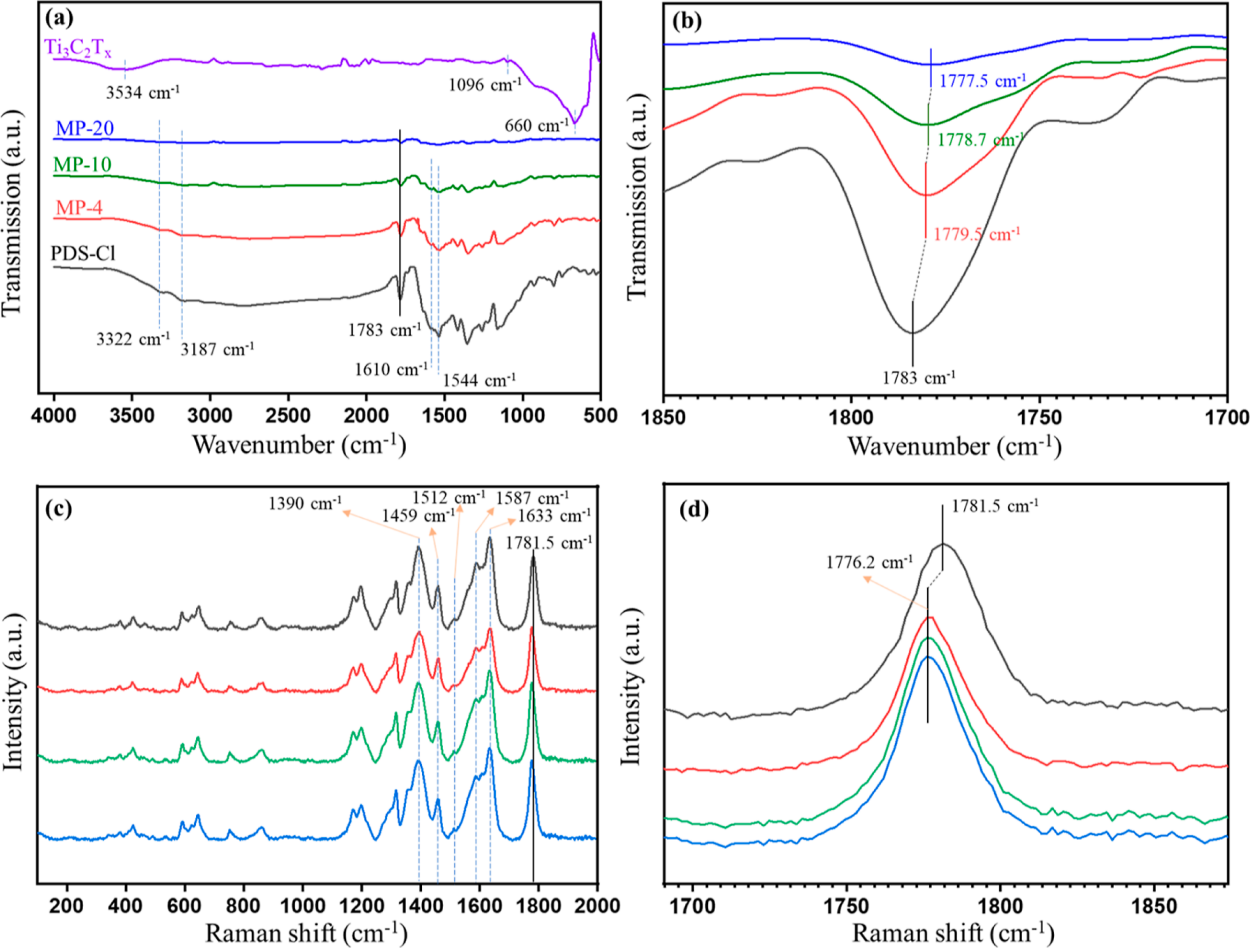
FTIR and Raman characterization: (a) FTIR spectra of pristine Ti_3_C_2_T_*x*_, PDS-Cl, and composite samples (MP-4, MP-10, and MP-20) with color coding and labeled characteristic features. (b) Redshift of the C=O peak in the FTIR spectra. (c) Raman spectra of pristine Ti_3_C_2_T_*x*_, PDS-Cl, and composite samples (MP-4, MP-10, and MP-20) along with (d) redshift of the C=O peak in Raman spectra.

Fourier transform infrared (FTIR) and Raman spectra of pristine Ti_3_C_2_T_*x*_, PDS-Cl polymers, and the Ti_3_C_2_T_*x*_/PDS-Cl composite, as shown in Figure 3, reveal the ionic nature of the PDS-Cl and the hydrogen bonding between the MXene flakes and the polymers. For a better comparison of FTIR and Raman spectra, we have only selected 3 composite samples (out of 6 samples utilized for gas sensing) with mass ratios of 4, 10, and 20 wt % denoted as MP-4, MP-10, and MP-20, respectively. In the FTIR spectrum for the PDS-Cl, as shown in Figure 3a, the peaks at 3322 and 3187 cm^–1^ are attributed to the N–H group. The feature at 1783 cm^–1^ can be attributed to the cyclobutene carbonyl compound (C=O),^32^ which overlaps with expected C=N (imino) resonances at ca. 1780 cm^–1^. The peak at 1544 cm^–1^ originates from the C=C stretching vibrations of four-membered ring while the characteristic strong absorption peak at 1610 cm^–1^ in PDS-Cl can be assigned to the vibration peak of the aromatic structure and zwitterionic resonance of the cyclobutene 1,3-diolate anion moiety, indicating the successful synthesis of the PDS-Cl with the anticipated structure, as shown in Figure 1a. The FTIR spectra of composites are almost similar to the PDS-Cl, except for the C=O stretching vibration (1783 cm^–1^), which gradually redshifts from 1783.0 cm^–1^ in PDS-Cl to 1777.5 cm^–1^ in MP-20 (Figure 3b). This redshift probably originates from the electron density redistribution due to extra hydrogen bonding from the Ti_3_C_2_T_*x*_ flakes.^33−35^ For Ti_3_C_2_T_*x*_, the peaks at 3534, 1096, and 668 cm^–1^ can be attributed to the stretching vibrations of −OH, C–F, and Ti–O bonds, in agreement with the previous reports.^36^

Raman spectra of PDS-Cl and composite samples also show a redshift (∼5 cm^–1^) for the C=O peak at 1781.5 cm^–1^, see Figure 3c,d. This spectrum also provides additional information about the PDS-Cl structure with the characteristic features attributed to the cyclic C=C stretching frequencies of cyclobutene (at 1633 cm^–1^) and the quadrant and semicircle stretching vibrations of the aromatic ring at 1587 and 1512 cm^–1^, respectively. Moreover, the vibration peaks of N^+^–CH_3_ and C–N^+^ (at 1459 and 1390 cm^–1^, respectively) imply the ionic nature of PDS-Cl.^37,38^

Although the FTIR spectrum of pristine Ti_3_C_2_T_*x*_ exhibits few characteristic features, as shown in Figure 3a, the Raman spectrum of thin films of Ti_3_C_2_T_*x*_ and MP-10, dispersed on a Si substrate, indicates several features, as shown in Figure S4. Spectra show peaks at 123, 202, and 723 cm^–^, which are assigned to the plasmonic resonance, the out-of-plane vibration of Ti, C, and surface group atoms, A_1g_ (Ti, C, and O), and the out-of-plane vibration of carbon atoms, A_1g_(C), respectively. The intensity ratio of A_1g_(C)/A_1g_ (Ti, C, O) varies depending on the sample.^39^ For instance, this ratio for the pristine sample is around 0.86, which implies a strong A_1g_ (Ti, C, and O) vibration as whole flakes while for composites, it increases to 1.2 as a sign of weak coupling between flakes due to intercalation of polymers. Moreover, the A_1g_ (Ti, C, and O) peak for composite shifts to lower wavenumbers, ∼198 cm^–1^, further indicating the disorder between the layers.^39^

**Figure 4:**
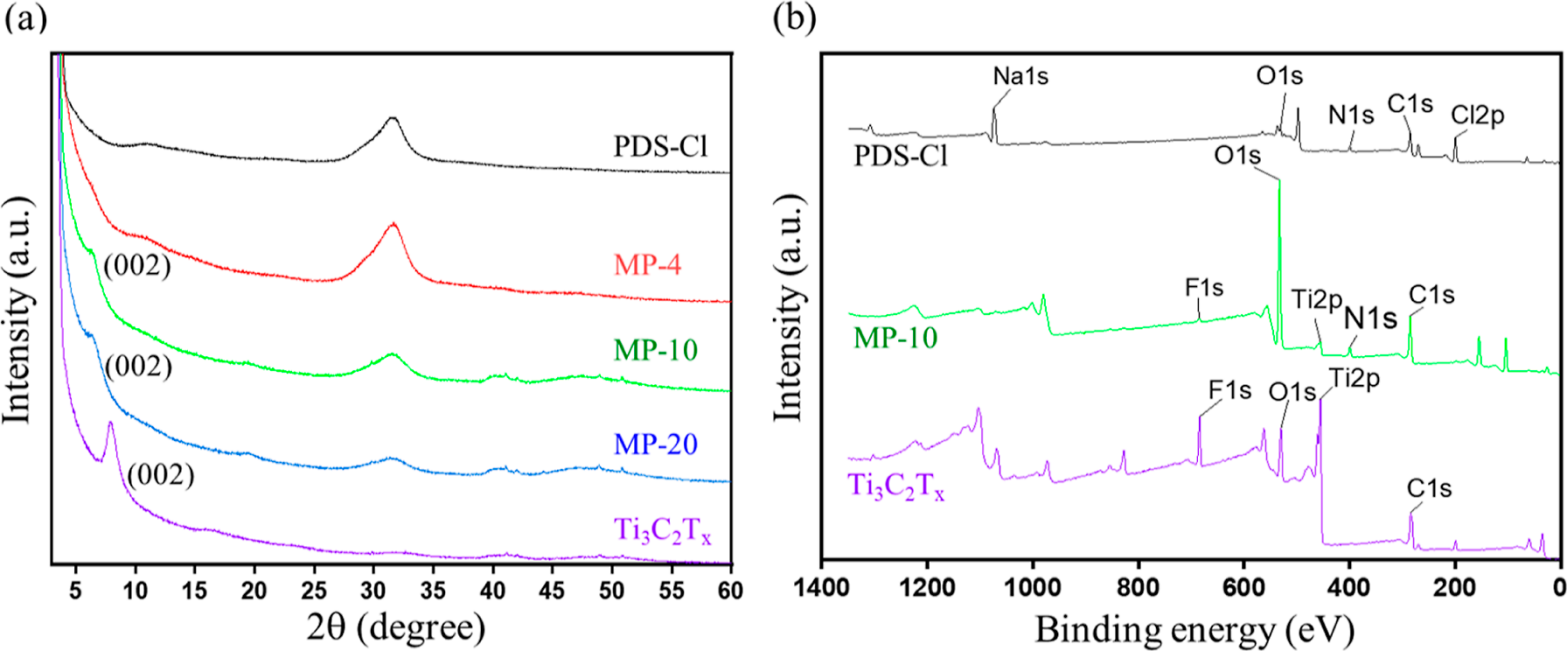
XRD and XPS analysis: (a) XRD spectra of pristine Ti_3_C_2_T_*x*_, PDS-Cl, and composite samples. (b) Survey spectrum of Ti_3_C_2_T_*x*_, PDS-Cl, and MP-10.

XRD measurements also support the disorder between the Ti_3_C_2_T_*x*_ flakes in the composites, as shown in Figure 4a. The characteristic reflection of the pristine Ti_3_C_2_T_*x*_ at 8° (11.0 Å) shifts to 6.4° (13.8 Å) for MP-10 and MP-20 along with peak broadening and disappears for the MP-4 sample, implying increased interlayer separation and a disorder in the stacking of the Ti_3_C_2_T_*x*_ flakes due to intercalation of PDS-Cl between layers.^40^

The functional groups, such as fluorine and oxygen, at the surface of the Ti_3_C_2_T_*x*_ flakes and composite samples were evaluated using XPS. Figure 4b shows the survey spectrum of Ti_3_C_2_T_*x*_, PDS-Cl, and MP-10, indicating the presence of those functional groups on the surface of the composite. The C 1s and F 1s spectra in Figure S5 indicate the interaction of carbon and fluorine in the MP-10 sample.^41^ In the F 1s spectrum of the pristine MXene (Figure S5b), the peaks at 684.5 and 685.2 eV are assigned to F–Ti and C–Ti–F_*x*_, respectively,^42,43^ whereas the F 1s spectrum for MP-10 contains a third peak at 689.65 eV, suggesting the presence of the interaction between carbon and fluorine with high binding energy.

### 3.2 Gas Sensing Performance

The gas sensors were fabricated by drop-casting equal amounts (1 μL of solution with concentration of 1 mg•mL^–1^) but different mass ratios of the MXene/PDS-Cl composite onto SiO_2_ (300 nm)-Si substrates. To find out the optimum mass ratio with the highest sensing response, we measured the H_2_S response of composite samples with 6 different mass ratios of 4, 6, 8, 10, 15, and 20 wt %. Figure S6 shows the base resistance of the Ti_3_C_2_T_*x*_/PDS-Cl composite samples, prior to gas sensing. A percolation threshold of φ_0_ = 6.00 ± 1.10 wt %, obtained by Belehradek power function fitting, indicates that all samples, other than 4%, contain conductive networks of MXene flakes.

The gas response is calculated using eq 2
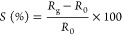
2where *R*_0_ and*R*_g_ are the resistances of the sensor upon exposure to N_2_ and the target gas, respectively.

**Figure 5:**
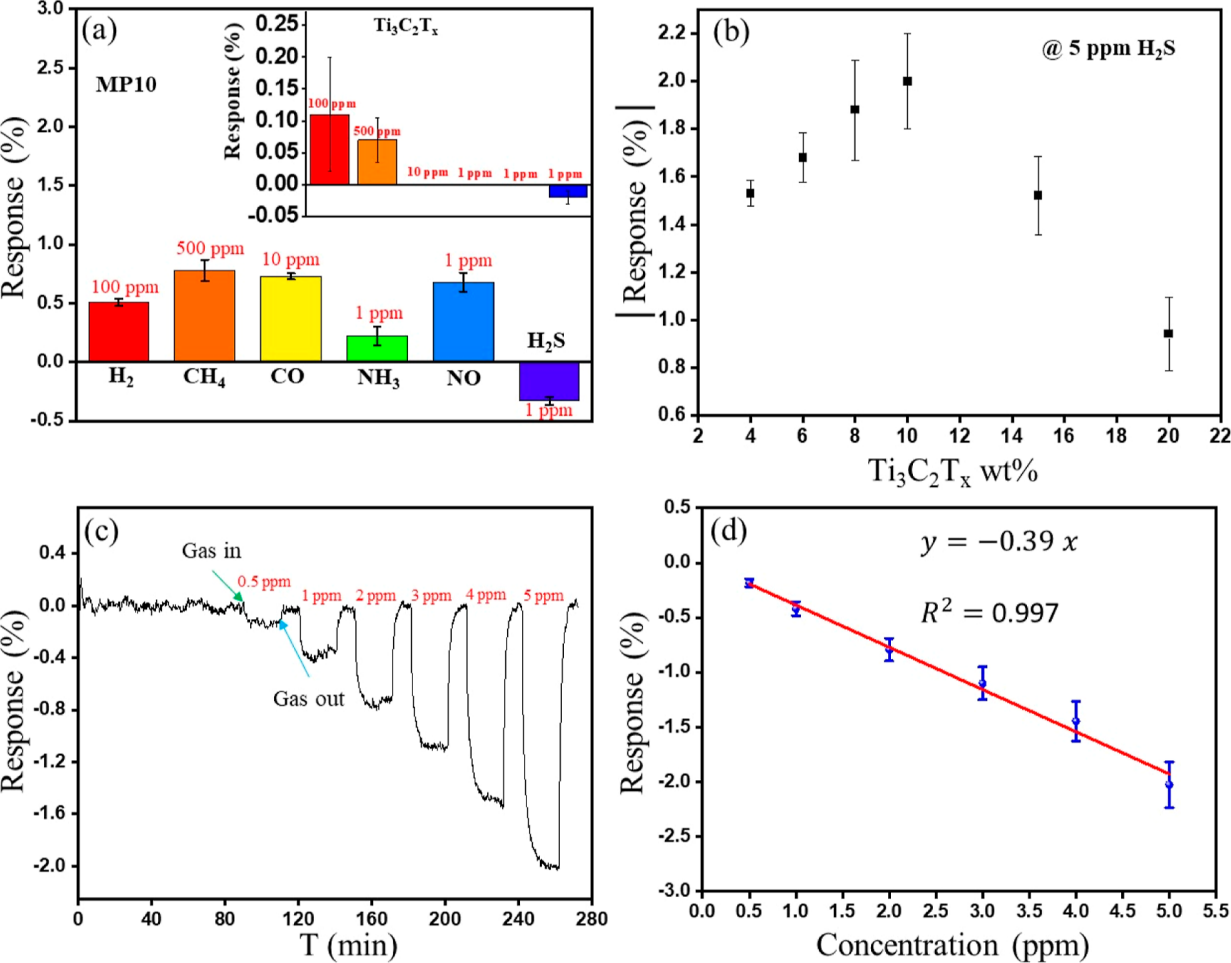
Selectivity and sensing response: (a) selectivity of MP-10 toward 6 different analytes with a negative response for H_2_S. The inset shows the selectivity of pristine Ti_3_C_2_T_*x*_. (b) H_2_S sensing response for the composite samples with different mass ratios of Ti_3_C_2_T_*x*_ in which the 10 percent shows the highest response. (c) Dynamic sensing response of MP-10 for different concentrations of H_2_S, with the baseline subtracted for clarity and readability of the signal. (d) Sensing response of MP-10 sample versus H_2_S concentration from 0.5 to 5 ppm (error bars indicate standard errors that are calculated using the data measured from three samples).

Figure 5a summarizes the selectivity responses from the MP-10 sample, and the results from pristine MXenes, in the inset. The composite sensor shows striking selectivity toward the H_2_S gas, with a negative response, while it is positive for all other analytes (see Figure S7 for real-time resistance curve of the sensor). Notably, the selectivity to H_2_S is already present in the pristine MXene sensor, although the response is much smaller as shown in the inset of Figure 5a and Figure S8a.

Among all the samples, MP-10 shows the highest sensing response, around 2 ± 0.2% at 5 ppm of H_2_S gas, as shown in Figure 5b, with a 30-fold increase of response at 1 ppm concentration of H_2_S compared to the pristine Ti_3_C_2_T_*x*_ (see Figure S8b). However, the noise level in MP-10 is higher compared to pristine Ti_3_C_2_T_*x*_ in Figure S8b because the polymer alters the charge transport between layers. The real-time resistance curves of gas sensing for all samples (with different wt % of Ti_3_C_2_T_*x*_) are shown in Figure S9.

Figure 5c shows the dynamic sensing response of the sensor for different concentrations of H_2_S where the baseline is subtracted for clarity and readability of the signal. Despite the long pre-measurement exposure to N_2_, around 1 h, the baseline is drifting; therefore, to calculate the correct response, a baseline curve, *R*_0_ has been fitted and subtracted from the data. The sensor response is a linear function of the gas concentration with a low LOD as low as 0.5 ppm, see Figure 5d. To ensure the result for LOD, we took 40 data points at the baseline before the H_2_S exposure in Figure 5c and calculated the noise level using the variation in the relative resistance change by the root-mean-square deviation.^44^ The absolute value of the response for MP-10 (0.14%) is at least five times higher than the noise level (0.023%), confirming a LOD of 0.5 ppm. Finally, we note that the sensor response toward humidity was also positive, as shown in Figure S10, with enhanced responsivity, compared to pristine Ti_3_C_2_T_*x*_.

**Figure 6:**
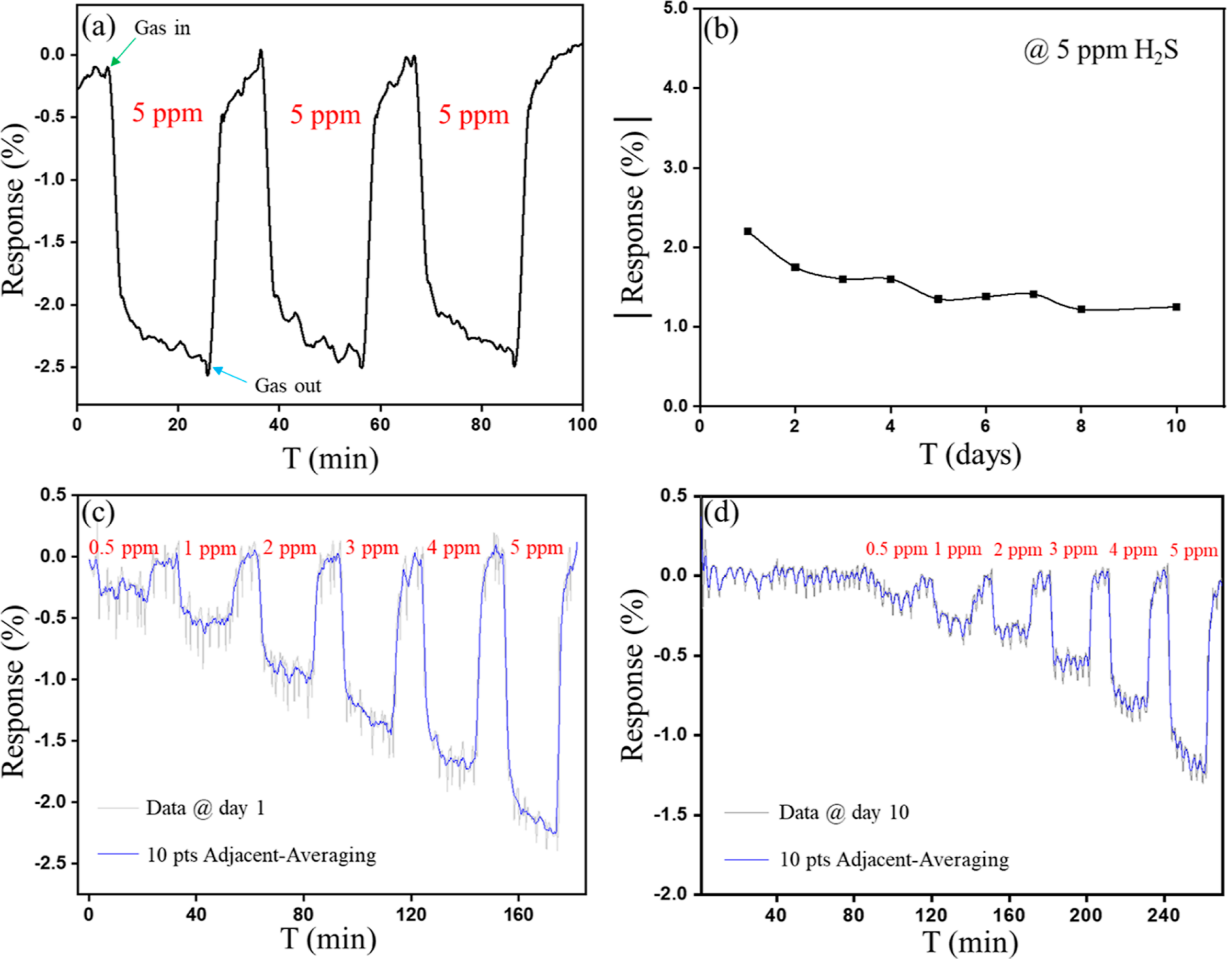
Repeatability and stability of sensor: (a) three cycles of MP-10 sensor response to 5 ppm H_2_S at room temperature. (b) Stability of MP-10 sensor to 5 ppm H_2_S over 10 days. The smoothed response–recovery curves of MP-10 sensor in day (c) 1 and (d) 10.

The sensor shows very good repeatability with a small variation (standard deviation of 0.076) under consecutive exposure to the H_2_S gas and recovers back to its initial state after gas removal, as demonstrated in Figure 6a.

The sensor stability under continuous measurements indicates that the response drops from −2.2 to −1.25% for 5 ppm of H_2_S after 10 days, as shown in Figure 6b, indicating a 60% stability compared to the initial response. Figure 6c,d shows the sensor response to H_2_S with concentrations varying from 0.5 to 5 ppm at day 1 and 10, respectively. The stability data is noisy, which could originate from the measurement setup rather than the material because exposure to the H_2_S gas has no effect on the noise level. To improve the readability, the data have been smoothed with 10 points adjacent averaging method. Figure S11 demonstrates the real-time resistance curve of the sensor for H_2_S sensing on days 1 and 10, in which the base resistance of the sensor increases in the course of time. Further studies are required to understand and enhance the stability, which might originate from the Ti_3_C_2_T_*x*_ oxidation and can be suppressed by modifying the device preparation or structure.^13^ In order to test the sensor’s performance for real-world applications, we also measured the H_2_S sensing performance of the MP-10 in an N_2_/O_2_ (80/20%) background, as shown in Figure S12, which indicates almost identical performance of the sensor.

### 3.3 H_2_S Gas Sensing Mechanism

The pronounced H_2_S selectivity may seem surprising in light of the previous results reported in the literature showing a positive response to all analytes. Pristine Ti_3_C_2_T_*x*_ films were studied in refs (9)(10)(45), and (48) and the general trends in the sensitivity agree with our results: sensitivity to ammonia and other “reactive” gases was high, but low to gases such as CH_4_ and CO_2_. In refs (10) and (45), all gases showed a positive response, but H_2_S was not included in these studies. Wu et al. measured the H_2_S response, but they only reported |Δ*R*|/*R*, i.e., the sign of the response is unknown, and the gas concentration was very high (500 ppm).^45^ Thus, although most papers have reported a positive response to any gas, the understanding of H_2_S response is, in fact, limited.

Using in situ XRD, Koh et al. found that the positive response of “reactive” gases such as ethanol correlated with increasing interlayer separation of (Na-intercalated) Ti_3_C_2_.^46^ This suggests a sensing mechanism, where the gases are intercalated between the layers and the resistivity increases due to the increasing interlayer separation of the conductive MXene sheets. Such a mechanism could explain the response to all gases with a positive response and the enhanced gas response in the MXene/polymer composite. However, the negative response (increasing conductivity) to H_2_S requires an inherently different mechanism. Also, the linear I–V characteristics of MP-10 (Figure S13) suggest ohmic contact between the sensing film and the electrodes; therefore, the H_2_S exposure modulates the conductivity of composites rather than contact resistance.

To gain insights into the possible sensing mechanism, we turned to atomistic modeling and DFT calculations. Because the negative response for H_2_S was recorded already for pristine Ti_3_C_2_T_*x*_ samples, we are looking for a mechanism that does not require the polymer but can still be enhanced by it. The majority of previously reported calculations have only considered pure O-terminated or pure OH-terminated surface.^10,45,47,48^ However, it is known from NMR and neutron/X-ray scattering experiments that the surfaces contain a mixture of O, OH, and F groups,^49,50^ and this functionalization is stable in vacuum or N_2_ atmosphere.^51^ In order to properly describe the interaction between the gas molecule and MXene surface, we thus adopt a model which contains a mixture of O, OH, and F groups in the O_0.50_OH_0.25_F_0.25_ composition. The adopted model was found based on our previous investigation, reflecting typically reported compositions.^52^ A significant concentration of O and F in our samples was also verified by EDX and XPS (Figures 2c and 4), although we cannot estimate the H concentration (i.e., the O/OH ratio) based on these methods. We note that gas adsorption on mixed-group surfaces of Ti_3_C_2_T_*x*_ was studied by Khakbaz et al.,^53^ but (i) these results were not compared to those from pure terminations and (ii) there was no detailed comparison to experimental results.

**Figure 7:**
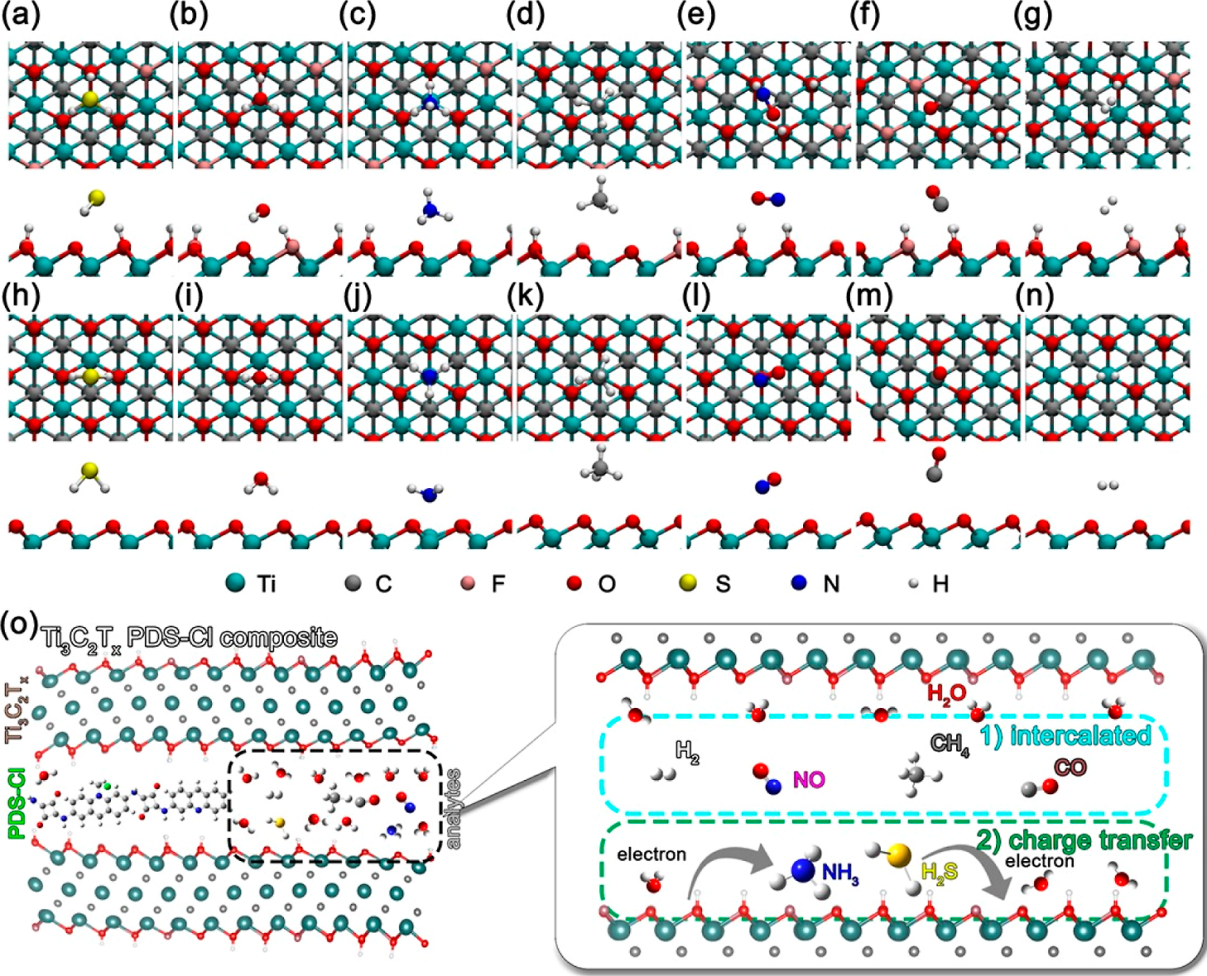
Adsorption geometries for different molecules on different MXene terminations: Panels (a–g) show the adsorption on a mixed surface (O_0.5_F_0.25_OH_0.25_) for H_2_S, H_2_O, NH_3_, CH_4_, NO, CO, and H_2_ molecules, respectively. Panels (d–n) show the adsorption on a pure O-terminated surface for the same respective molecules. Every panel has two parts: the top shows a top view of the MXene layer, while the bottom shows a side view of the MXene layer. Legend of the atom colors can be found at the very bottom of the figure. Panel (o) is the scheme of the proposed gas-sensing mechanism in which intercalation and charge transfer play the main role.

The charge transfers and adsorption energies of various gases on the pure O-terminated surface and the mixed-group surface are given in Table 1 and the adsorption geometries are depicted in Figure 7. The results are not only quantitatively but also qualitatively different owing to the larger variety of possible adsorption sites on the mixed surface and the different work functions (comparison of adsorption energies and charge transfer for similar sites are given in Table S2). We particularly note that H_2_S and H_2_O bind very strongly to mixed surfaces (much stronger than to the pure O-terminated surface) because the S atom of H_2_S and O atom of H_2_O can bind to the OH group and the H atoms to O groups. In fact, this result is fully consistent with reports of interlayer and surface water in MXenes (as observed, e.g., in TGA experiments),^54^ arising from its high hydrophilicity.^55^ NH_3_ appears to have even stronger adsorption energy, although this situation is somewhat different. Because when NH_3_ is adsorbed on the mixed surface, it captures a proton from one of the surface OH-groups (and 0.288e), resulting essentially in NH_4_^+^ adsorbed on negatively charged MXenes. We note that similarly high adsorption energies and NH_4_^+^ formation have been reported for mixed surfaces of vanadium carbide.^56^ Such high adsorption energies would strongly favor analyte adsorption but rule out analyte desorption under ambient conditions, which is clearly not the case according to experiments. Instead, we propose that the omnipresent water will play a central role here in displacing the analytes from the surface, i.e., the analyte adsorption should rather be described by the adsorption energy difference with respect to H_2_O.

**Table 1:** Charge Transfers and Adsorption Energies for the Different Molecules When Adsorbed on the Mixed Surface and the Purely O-Terminated Surface^a^

	mixed surface		purely O-terminated surface	
molecules	charge transfer	adsorption energy (meV)	charge transfer	adsorption energy (meV)
H_2_	0.091	–270	0.069	–151
CH_4_	–0.005	–216	0.008	–197
CO	0.152	–550	0.116	–248
NH_3_	–0.288	–1349	0.316	–613
NO	–0.135	–448	0.321	–538
H_2_S	0.163	–905	0.168	–383
H_2_O	0.099	–1056	0.018	–263

a Positive values of the charge transfer represent electrons being transferred from the molecule to the surface. Only lowest energy configurations are shown, see Table S3 for the full version that includes results from other configurations.

The markedly higher work function of the O-terminated surface (calculated to be 6.17 eV^52^ vs 4.3 eV for the mixed surface adopted here, Figure S15) is expected to lead to larger electron transfer from the analyte to MXene, i.e., more positive values for charge transfer in Table 1. This is indeed the case of CH_4_ and NO on the O-site and H_2_S on the O-site (Table S3). However, these are not the lowest energy configurations for NO and H_2_S, and when the analyte is bonded to OH groups the charge transfer can be qualitatively different.

In light of the above discussion, two mechanisms are likely at play: (1) the intercalation-induced increase of the interlayer separation and (2) charge transfer-induced modifications in carrier density. Mechanism 1 will contribute to all analytes, but it will dominate whenever the adsorption energy of the analyte cannot compete with the H_2_O adsorption and certainly includes H_2_ and CH_4_, perhaps also CO and NO. In all these cases, the response is positive irrespective of the direction of the calculated charge transfer. Mechanism 2 can dominate over mechanism 1 in the case of H_2_S and NH_3_ for which the adsorption energy is comparable or higher than that of H_2_O. In order to estimate how the charge transfer would affect resistance, instead of ballistic conductance calculations,^57,58^ we here rely on ref (59) where it was computationally shown that when O content of Ti_3_C_2_T_*x*_ surfaces is more than 50%, an increasing electron concentration (positive charge transfer from the analyte) leads to increasing conductivity and thereby a negative sensor response,^59^ as is the case with H_2_S.

The response of NH_3_ is more complicated, which makes it difficult to draw firm conclusions based on the calculations. Because NH_3_ captures H^+^ and 0.29e from MXenes, and H atoms in the OH group contains about 0.8e, 0.51e remains in the MXene and the response should be in the same direction as with H_2_S, in contrast to the experiments. On the other hand, when abundant water is present, as discussed above, NH_3_ can also capture protons from them, and then NH_4_^+^ adsorbed on the mixed surface is expected to accept ∼0.29e from the MXene. H^+^ capture was not found in the calculations in ref (53) but found in ref (56) for vanadium carbide suggesting that it is sensitive to the surface composition. Furthermore, even though our films are thin, there are still multilayer MXene flakes as evidenced by XRD and the positive response from the intercalation might overcome the charge-transfer effect. After all, the adsorption energy of NH_3_ (or NH_4_^+^) is very high and thus readily intercalated. The relatively slow recovery time of NH_3_ (Figures S7 and S14) also points to a strong interaction.

In the case of MXene/PDS-Cl composites, the response is enhanced while the selectivity is preserved. As the polymer opens the interlayer spaces, the number of accessible active sites increases and the intercalation becomes easier. This can lead to enhancement of the charge-transfer effect (mechanism 2). As for mechanism 1, the situation is less clear: a larger concentration of intercalated analytes between the sheets can enhance the effect, while the induced changes in the interlayer separation might be reduced due to initially larger spacing. A final contribution arises from the geometrical effect, wherein the number of conductive paths is reduced with increasing polymer content (or decreasing the wt % of Ti_3_C_2_T_*x*_; see resistance in Figures S5 and S8), which makes the sensor more sensitive (and noisy). On the other hand, the number of adsorption sites is expected to increase with increasing polymer content, and therefore there should be a trade-off for response as Figure 5b indicates. As the PDS-Cl/CNT composite also exhibits a small negative response toward H_2_S whereas bare CNTs do not, see Figure S15. We cannot rule out a synergic effect based on the adsorption of analytes on the PDS-Cl. However, we note that the conductivity of bare PDS-Cl samples was below the detection limit of our instruments and according to our calculations, the MXene Fermi-level resides between the frontier orbitals of PDS-Cl (Figure S16) consistent with small conductivity.

## 4 Conclusions

In summary, we have observed high selectivity in pristine thin films of Ti_3_C_2_T_*x*_ toward H_2_S gas sensing. Utilizing conjugated PDS-Cl polymers, we could preserve the selectivity and enhance the gas-sensing response thirtyfold at 1 ppm. The optimized sensor with 10 wt % of MXenes indicated a response of 2% at 5 ppm with an LOD of 500 ppb. To shed light on the sensing mechanism, we carried out DFT calculations. We have accounted for the fact that MXene surfaces contain a mixture of O, OH, and F functional groups and show that this has a dramatic effect on the gas adsorption (charge transfer and adsorption energy). The experimentally observed trends could be reproduced relying on the analyte intercalation and the charge-transfer mechanism from adsorbed analytes in competition with water molecules. This report expands the MXene/organic heterojunction application and enhances the understanding of gas sensing mechanisms in MXene-based sensors.

## Supporting Information Available

Required mass ratio of Ti_3_C_2_T_*x*_ and PDS-CL for samples with different wt % of Ti_3_C_2_T_*x*_; schematic of gas sensing setup; DLS data of pristine MXene, MP-4, 10, and 20; EDX imaging of the composite sample; Raman spectra of pristine Ti_3_C_2_T_*x*_ and MP-10; XPS spectra for C 1s and F 1s; base resistance of composite sample with different weight ratios of Ti_3_C_2_T_*x*_ along with power function fitting; real-time resistance curve of MP-10 sensor for different analytes; selectivity and real-time resistance curve of pristine Ti_3_C_2_T_*x*_ for different concentrations of H_2_S; I–V measurement of MP-10; real-time resistance curves; charge transfers and adsorption energies for the different molecules calculated with PBEsol functional but without van der Waals corrections; charge transfers and adsorption energies for the different molecules when adsorbed on the mixed surface and the purely O-terminated surface; and energy levels of the PDS-Cl polymer compared to the Fermi level of mixed MXenes (PDF)
