# Supplementary material for: Highly Selective H2S Gas Sensor Based on Ti3C2Tx MXene–Organic Composites
Source: ACS Appl Mater Interfaces. 2023 Jan 25;15(5):7063–73. doi: 10.1021/acsami.2c19883 (PMC9923678; doi:10.1021/acsami.2c19883)
Supplement: Supplementary file 1 — am2c19883_si_001.pdf [file am2c19883_si_001.pdf]

Supporting Information

Highly selective H_2S gas sensor based on $Ti_3C_2T_x$ MXene-Organic composite

Seyed Hossein Hosseini Shokouh[a], Jin Zhou[a], Ethan Berger[a], Zhong-Peng Lv[b,]*, Xiaodan Hong[b], Vesa Virtanen[c], Krisztian Kordas[a], Hannu-Pekka Komsa[a,]*

[a] Microelectronics Research Unit, Faculty of Information Technology and Electrical Engineering, University of Oulu, P.O. Box 4500, FIN-90014 Oulu, Finland.
[b] Department of Applied Physics, Aalto University, FIN-00076 Aalto, Finland.
[c] Research Unit of Medical Imaging, Physics and Technology, Faculty of Medicine, University of Oulu, Aapistie 5A, 90220 Oulu, Finland.

Corresponding author: Hannu-Pekka Komsa and Zhong-Peng Lv
Email: Hannu-Pekka.Komsa@oulu.fi and zhongpeng.lyu@aalto.fi

Table S1: The mass ratio of $Ti_3C_2T_x$ and PDS-CL for samples with different wt% of $Ti_3C_2T_x$.

Concentration of $Ti_3C_2T_x$ ink (mg·mL^{-1})	Volume of $Ti_3C_2T_x$ ink (μL)	$Ti_3C_2T_x$ weight (μg)	PDS-Cl weight (μg)	Wt% $(w_{Ti_3C_2T_x}/w_{PDS-Cl})$*100
12	3.3	~ 40	1000	4
12	5.0	~ 60	1000	6
12	6.7	~ 80	1000	8
12	8.3	~ 100	1000	10
12	12.5	~ 150	1000	15
12	16.7	~ 200	1000	20

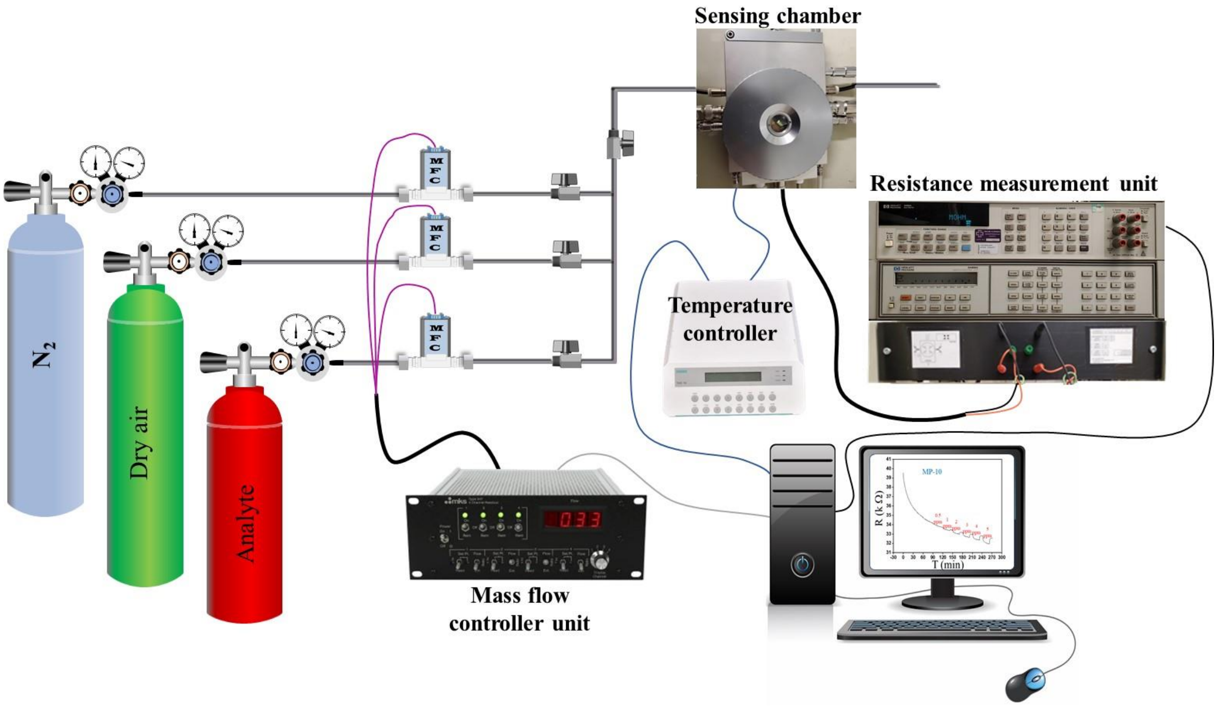

Figure S1: Schematic of gas sensing setup along with temperature controller, mass flow controller, and resistance measurement units.

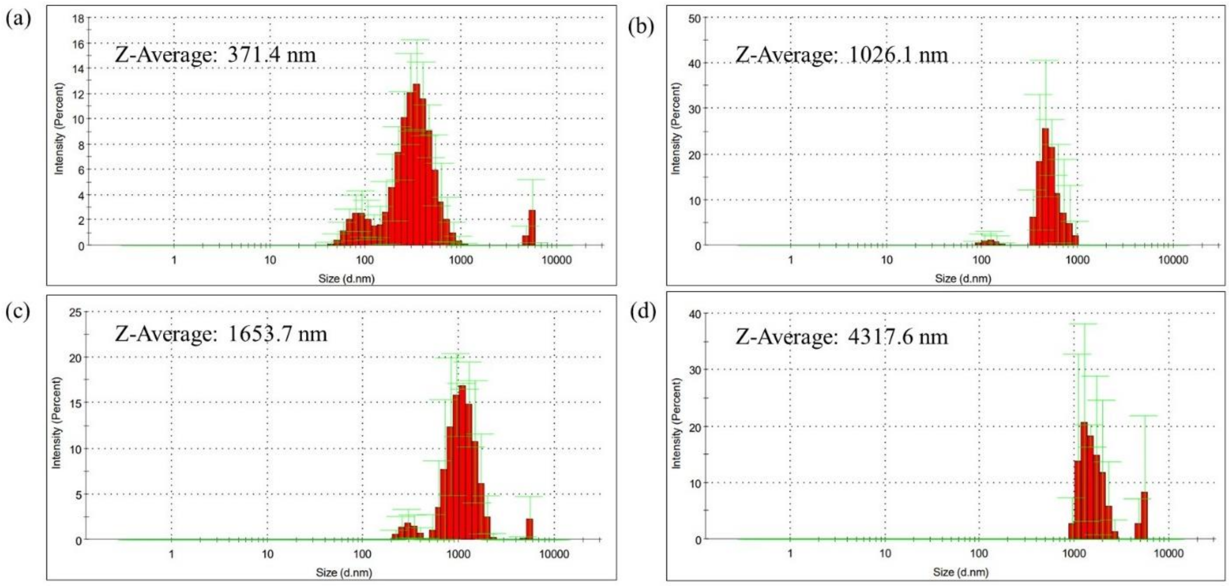

Figure S2: Dynamic light scattering (DLS) data shows the particle size distribution and average particle size for (a) Pristine $Ti_3C_2T_x$, (b) MP-4, (c) MP-10, and (d) MP-20.

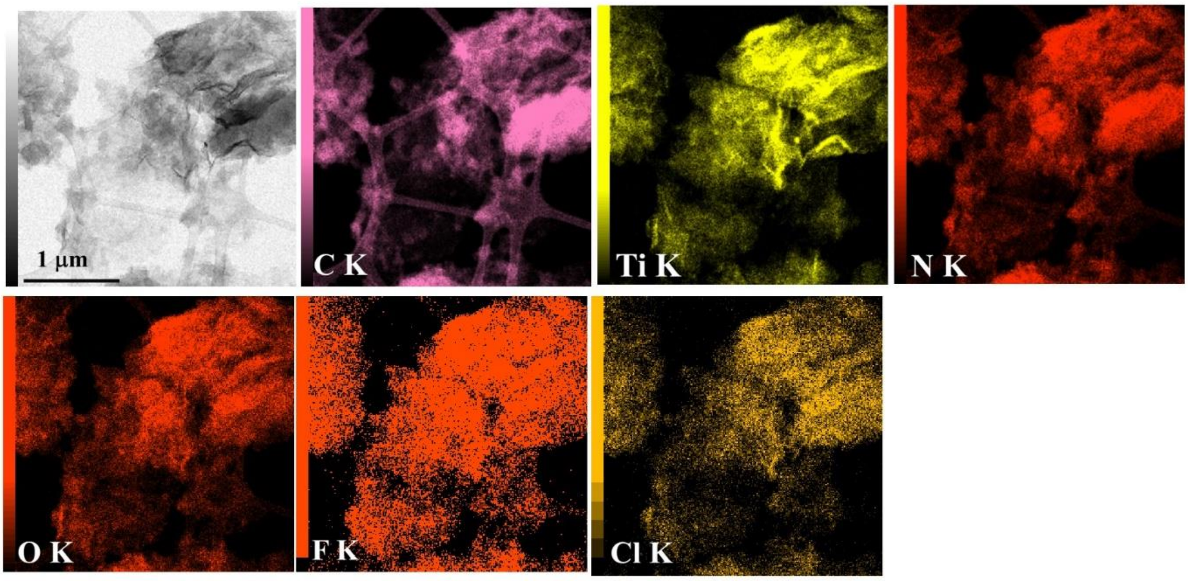

Figure S3: The EDX imaging of the composite sample (MP-10).

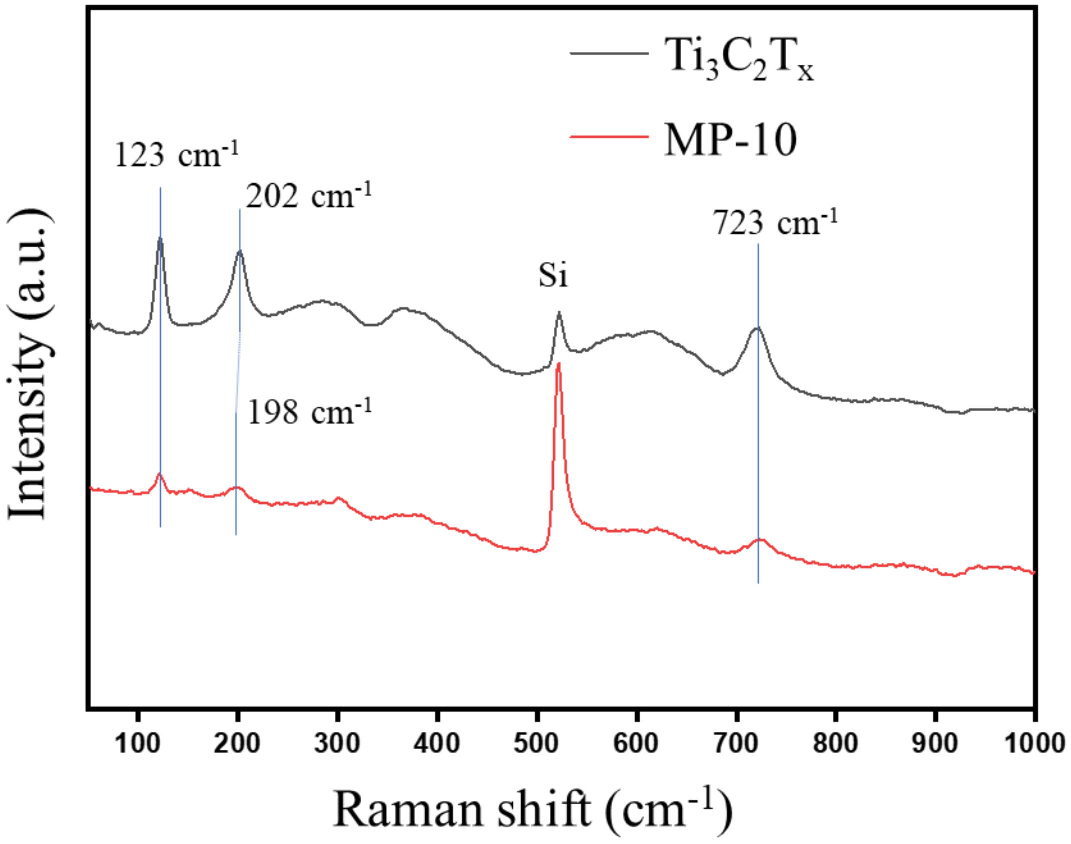

Figure S4: The Raman spectra of pristine $Ti_3C_2T_x$ and MP-10 with characteristic features.

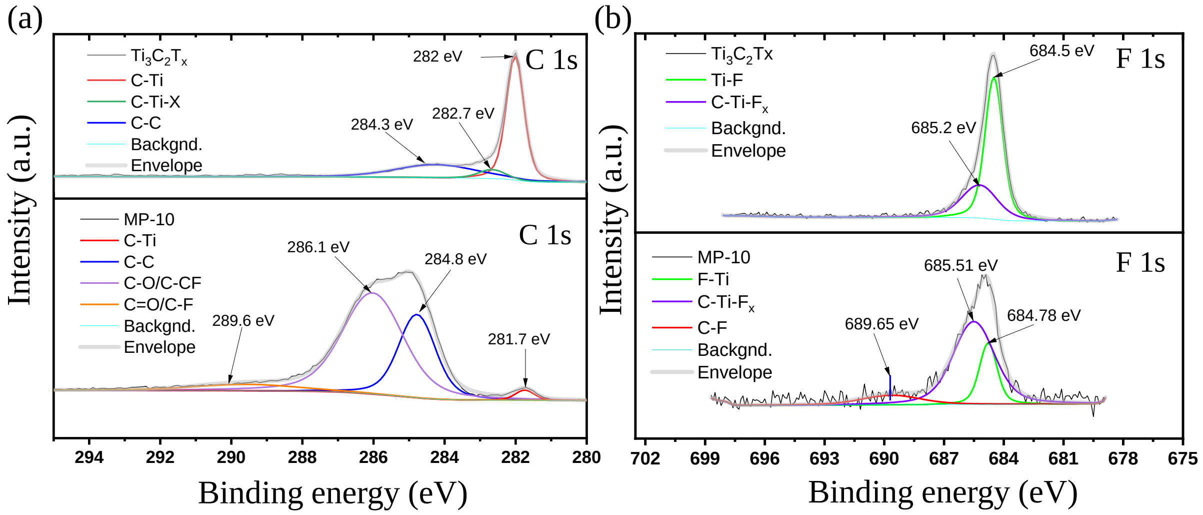

Figure S5: (a) XPS spectrum of C 1s of $Ti_3C_2T_x$ and MP-10 and (b) XPS spectrum of F 1s of $Ti_3C_2T_x$ and MP-10 along with peak positions and corresponding chemical bonds.

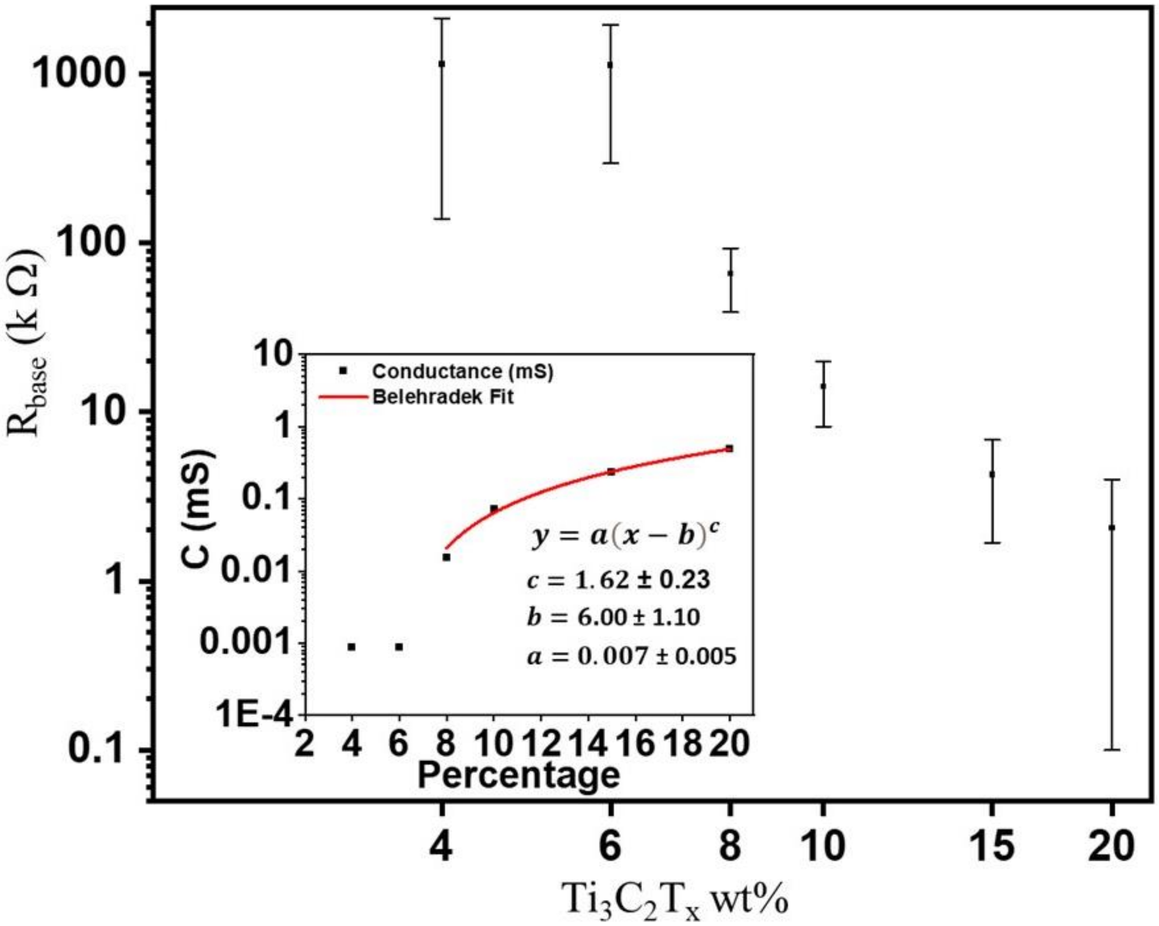

Figure S6: The base resistance of composite sample with different weight ratios of $Ti_3C_2T_x$. The inset shows the conductance of samples versus the MXene wt% with Belehradek power functions fitting that indicates a percolation threshold at around 6 wt%.

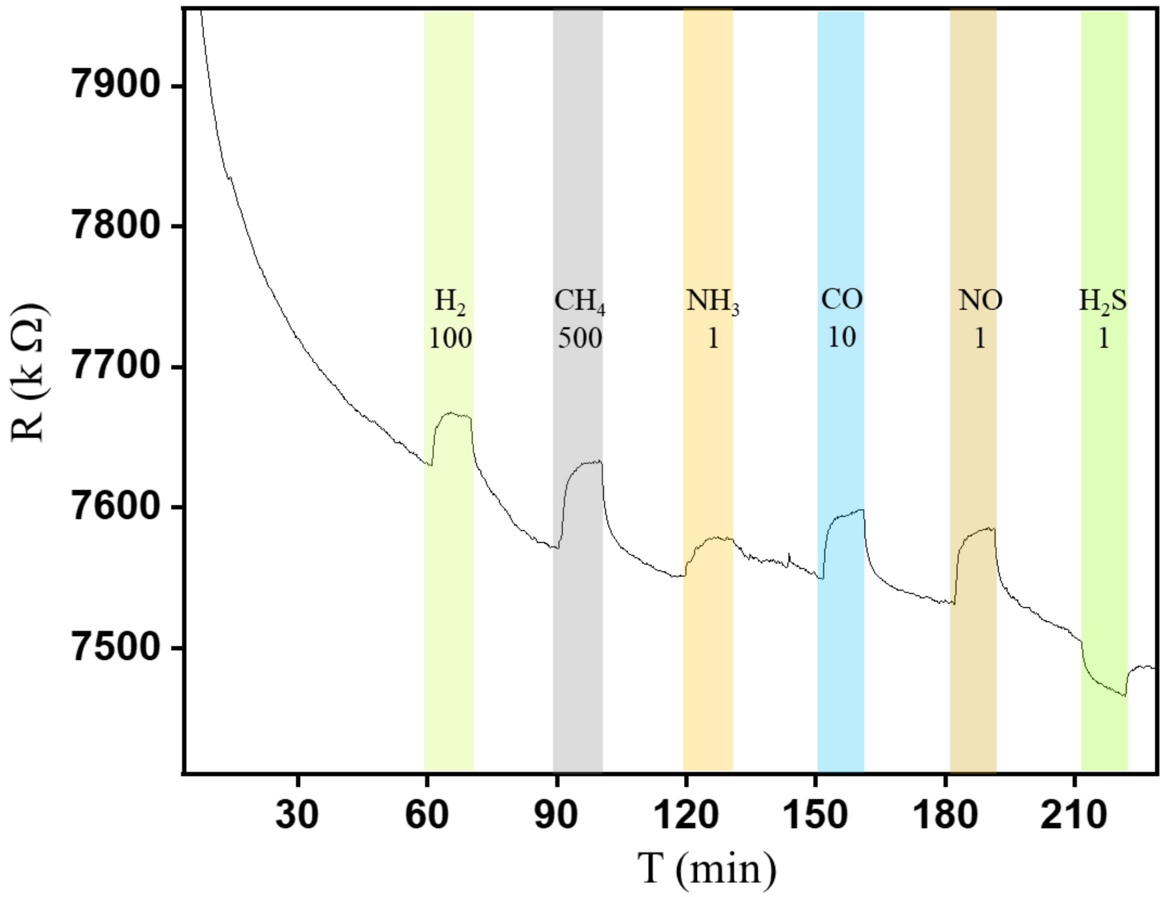

Figure S7: The real-time resistance curve of MP-10 sensor for different analytes.

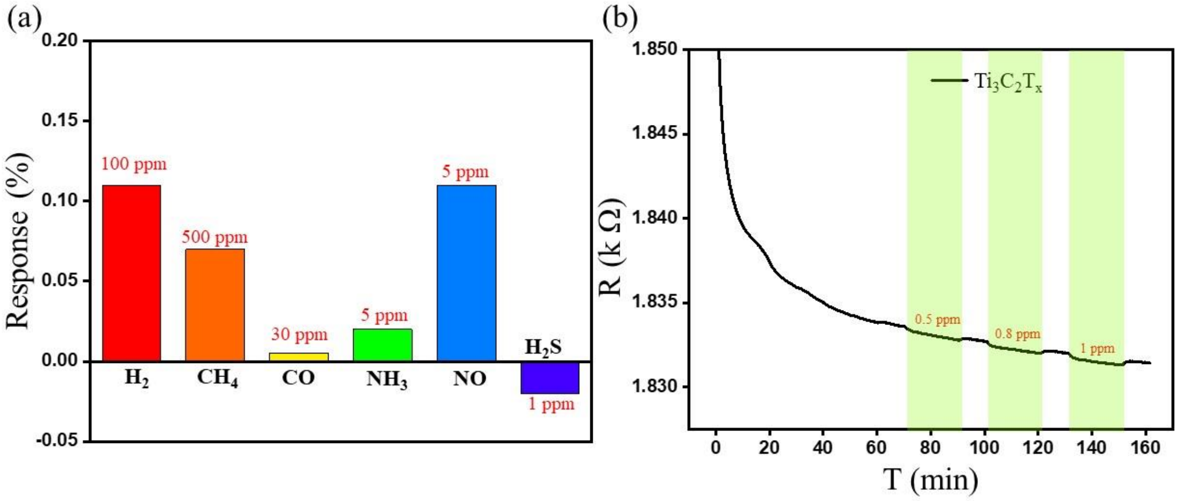

Figure S8: (a) The selectivity of pristine $Ti_3C_2T_x$ toward 6 different analytes with negative response for H_2S. (b) The real-time resistance curve of pristine $Ti_3C_2T_x$ for different concentrations of H_2S.

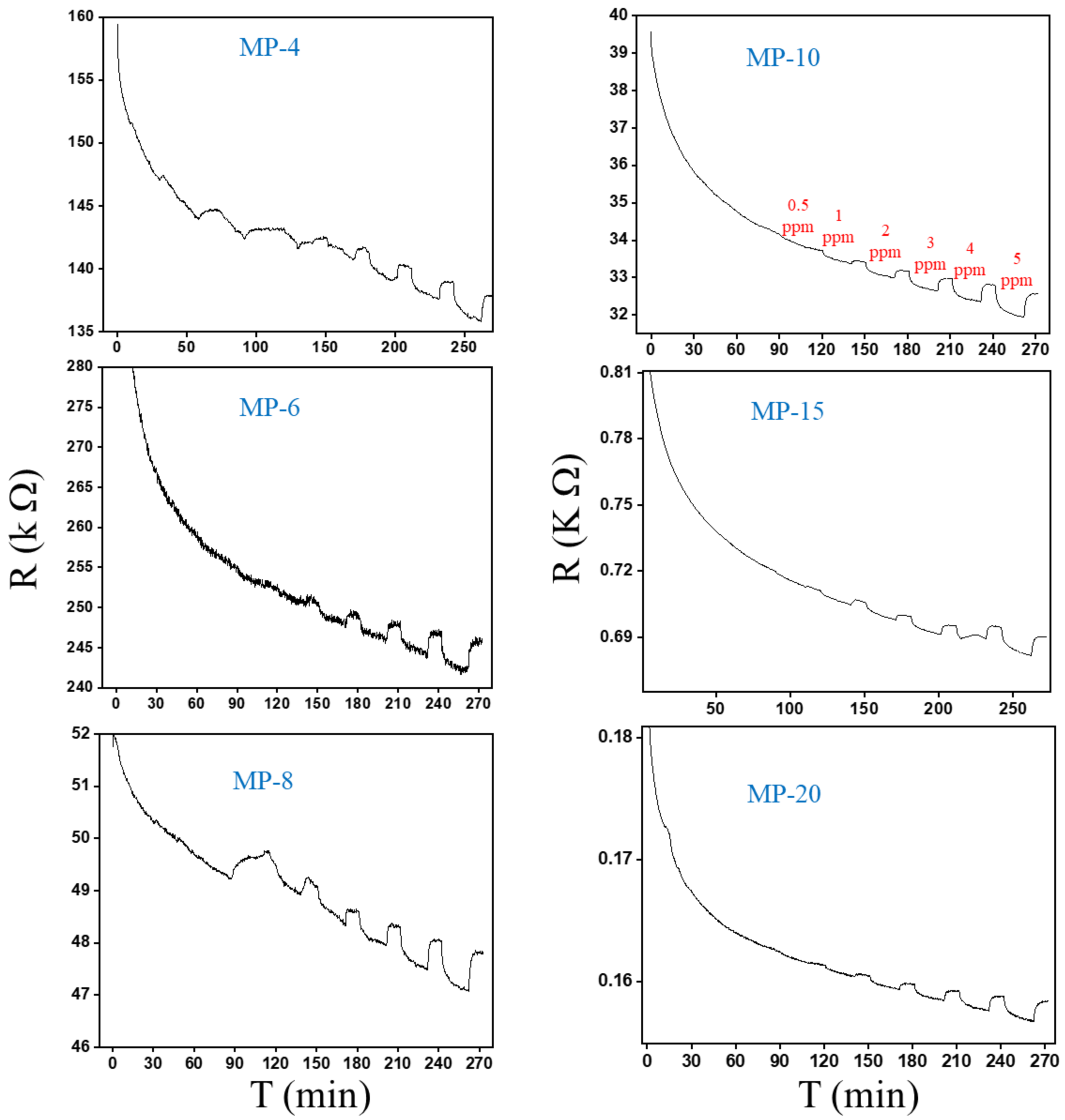

Figure S9: The real-time resistance curve of composite samples for different concentrations of H_2S gas from 0.5 to 5 ppm.

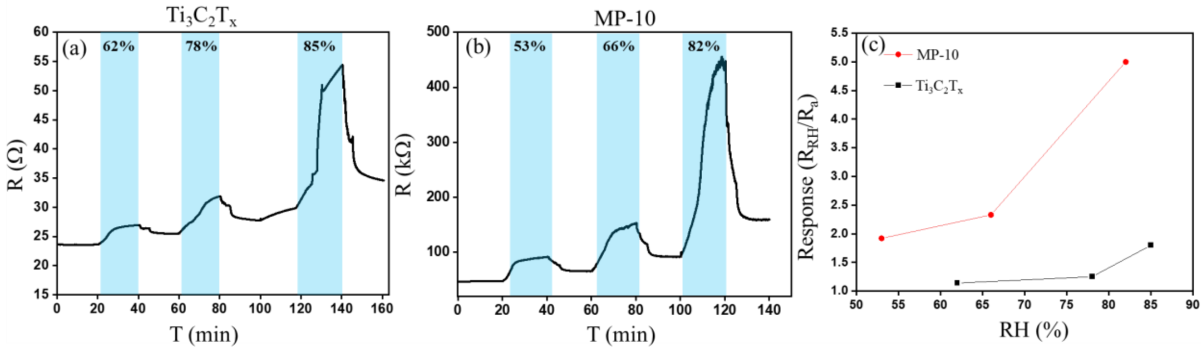

Figure S10: The real-time resistance curve of (a) pristine Ti_3C_2Tx and (b) MP-10 at the different levels of relative humidity (RH) at room temperature. (c) The humidity sensing response of pristine MXene and MP-10 versus the different levels of RH with the base level (R_a) of 20%.

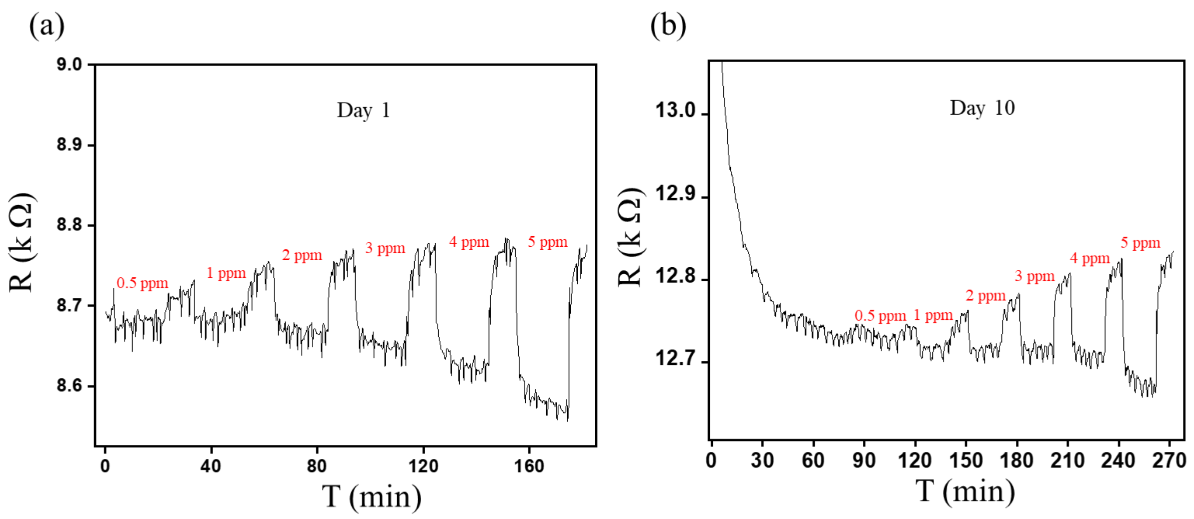

Figure S11: The real-time resistance curve of MP-10 sensor for H_2S sensing on the day (a) one and (b) ten.

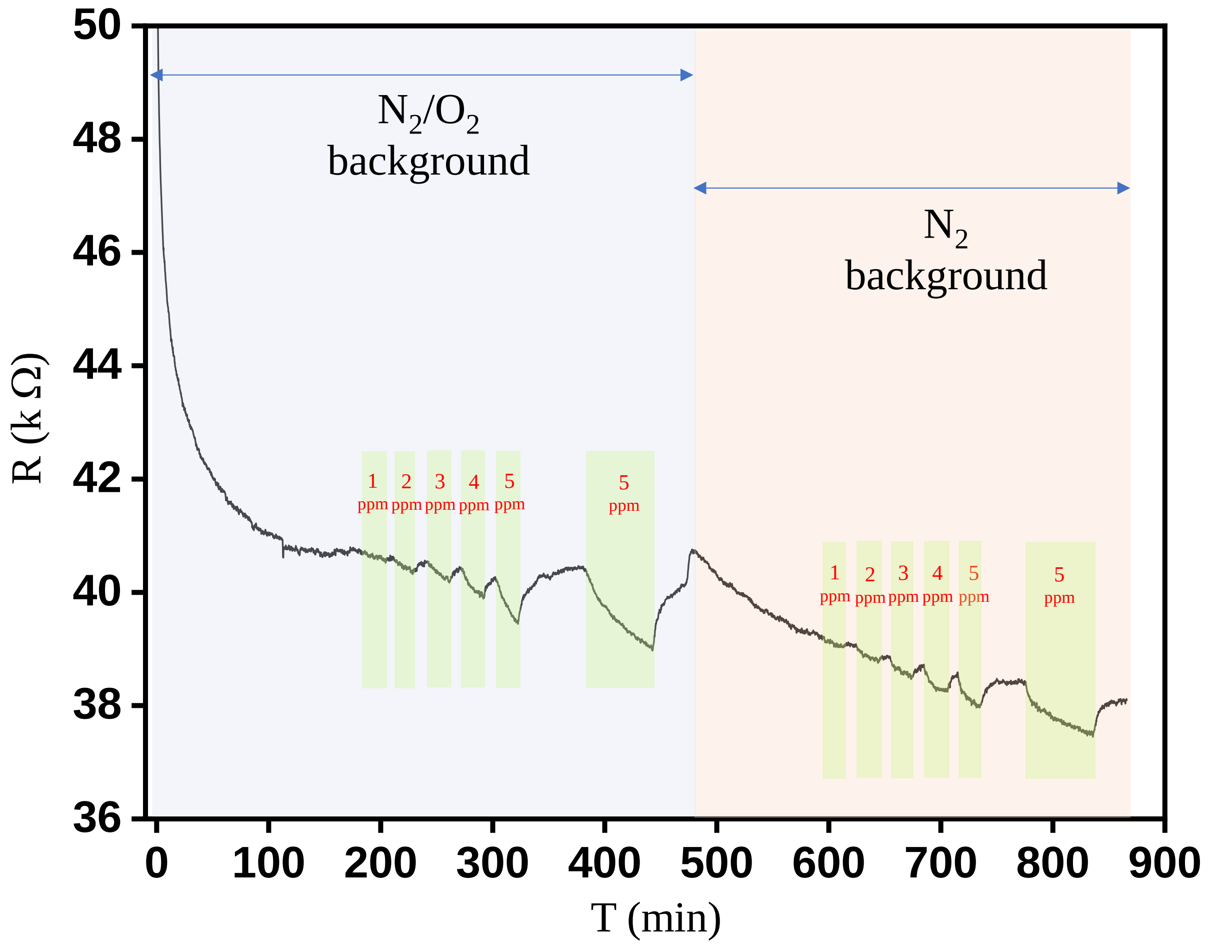

Figure S12: The real-time resistance curve of MP-10 sensor for different concentrations of H_2S gas from 1 to 5 ppm in N_2/O_2 and N_2 background.

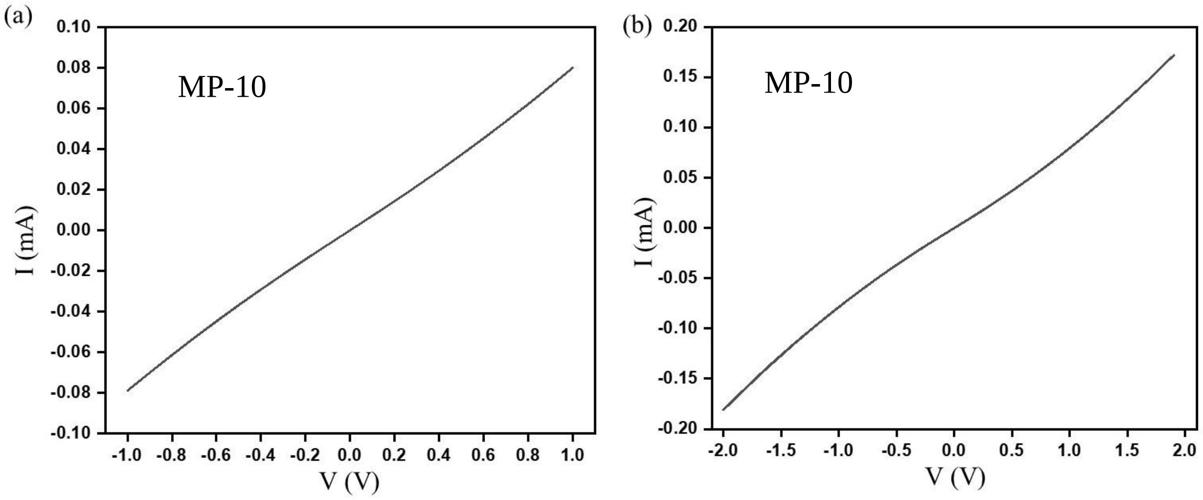

Figure S13: The I-V characteristic of MP-10 at (a) -1 to 1 V and (b) -2 to 2 V indicates an ohmic contact with electrodes.

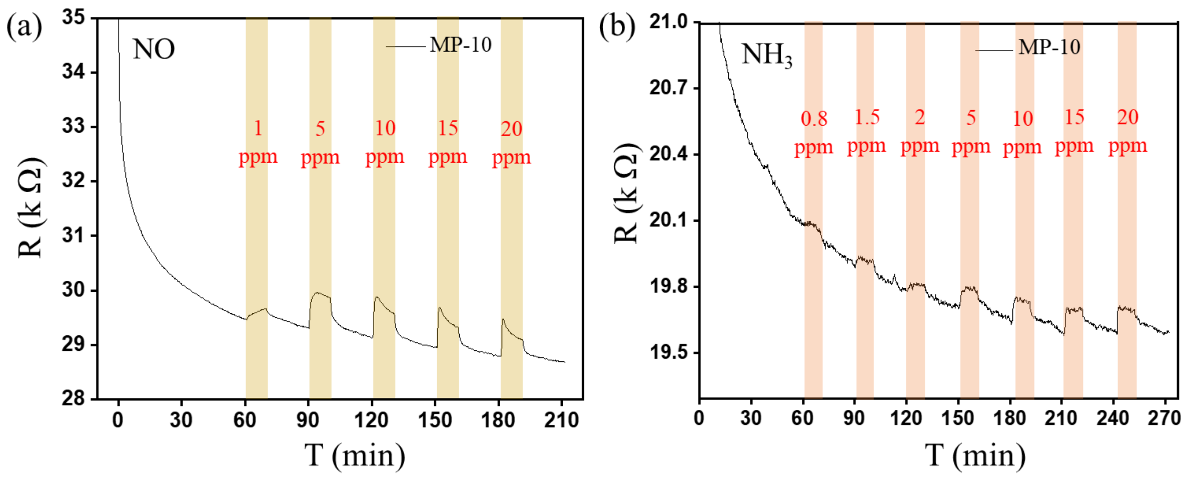

Figure S14: The real-time resistance curve of MP-10 sensor for different concentrations of (a) NO and (b) NH_3.

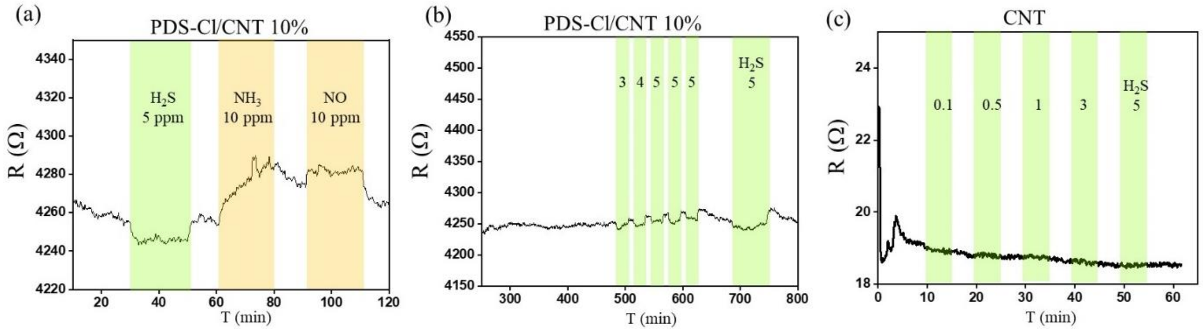

Figure S15: (a) The real-time resistance curve of PDS-Cl/CNT composite with 10 wt% conductive CNT for different analytes. The composite did not show any noticeable response to H_2, CH_4 and CO. (b) The real-time resistance curve of H_2S gas sensor based on PDS-Cl/CNT composite with 10 wt% conductive CNT and (c) pristine CNT. The response for the composite sample is negative (same as MXene composite but with a smaller value), while there is no response for the pristine CNT toward H_2S; therefore, the H_2S sensing response in PDS-Cl/CNT should originate from polymer.

Table S2: Charge transfers and adsorption energies for the different molecules when adsorbed on the mixed surface and the purely O-terminated surface calculated with PBE sol functional but without van der Waals corrections. Positive values of the charge transfer represent electrons being transferred from the molecule to the surface.

**Molecules**	**Mixed surface**		**Purely O-terminated surface**	
	Charge transfer	Adsorption energy (meV)	Charge transfer	Adsorption energy (meV)
**CH_4**	–0.004	–31	0.003	–27
**NH_3**	–0.281	–1115	0.308	–366
**NO**	–0.137	–310	0.318	–378
**H_2S**	0.164	–645	0.159	–117
**H_2O**	0.101	–869	0.019	–109
**CO**	0.150	–393	0.094	–119
**H_2**	0.090	–172	0.046	–54

Table S3. Charge transfers and adsorption energies for the different molecules when adsorbed on the mixed surface and the purely O-terminated surface. This is a full version of Table 1 in the main text showing also results for O-site adsorption in order to facilitate comparison to the purely O-terminated surface although not the lowest energy configurations. In many cases, the analyte placed on the O-site spontaneously moved next to OH groups.

**Molecules**	**Mixed surface**		**Purely O-terminated surface**	
	Charge transfer	Adsorption energy (meV)	Charge transfer	Adsorption energy (meV)
**H_2**	0.091	–270	0.069	–151
**CH_4**	–0.005	–216	0.008	–197
**CO**	0.152	–550	0.116	–248
**NH_3**	–0.288	–1349	0.316	–613
**NO (O-site)**	0.169	–283	0.321	–538
**NO (OH-site)**	–0.135	–448	-	-
**H_2S (O-site)**	–0.018	–394	0.168	–383
**H_2S (OH-site)**	0.163	–905	-	-
**H_2O**	0.099	–1056	0.018	–263

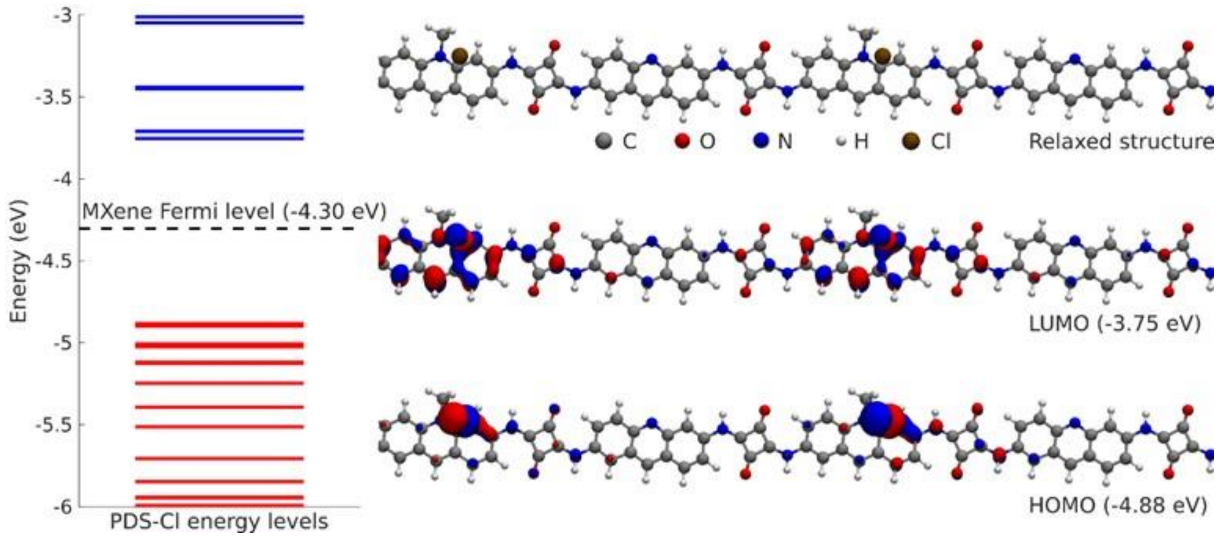

Figure S16: On the left, energy levels of the PDS-Cl polymer compared to the Fermi level of mixed MXene. On the right, representation of the relaxed structure as well as the molecular orbitals of HOMO and LUMO of the PDS-Cl polymer.
